# Refining silicon nitride waveguide quality through femtosecond laser annealing

**DOI:** 10.1038/s41598-024-66707-5

**Published:** 2024-07-08

**Authors:** Pei-Hsun Wang, Chien-Hung Chen, Nien-Lin Hou, Jia-Hao Cao, He-Yuan Zheng, Hung-Wen Chen

**Affiliations:** 1 Department of Optics and Photonics, National Central University, Taoyuan City, 320317 Taiwan; 2 International Intercollegiate Ph.D. Program, National Tsing Hua University, Hsinchu, 30013 Taiwan; 3 Institute of Photonics Technologies, National Tsing Hua University, Hsinchu, 30013 Taiwan

**Keywords:** Silicon photonics, Integrated optics

## Abstract

We present a method for modification of silicon nitride (Si_3_N_4_) waveguide resonators using femtosecond laser annealing. The quality (Q) factor of the waveguide resonators can be improved by approximately 1.3 times after annealing. Notably, waveguides that originally had a high Q value maintained their quality after the annealing process. However, those with a lower initial Q value experienced a noticeable improvement post-annealing. To characterize the annealing effect, the surface morphologies of Si_3_N_4_ films, both pre- and post-annealing, were analyzed using atomic force microscopy. The findings suggest a potential enhancement in surface refinement. Furthermore, Raman spectroscopy confirmed that the Si_3_N_4_ film's composition remains largely consistent with its original state within the annealing power range of 0.6–1.6 W. This research underscores the potential of femtosecond laser annealing as an efficient, cost-effective, and localized technique for fabricating low-loss integrated photonics.

## Introduction

Laser annealing is highly popular for advanced technology nodes in industrial applications^1–3^. Due to the rapid developments of micro- and nano-structural modifications of materials, laser annealing has become increasingly important in manufacturing processes. In comparison to the traditional thermal annealing through a furnace-based system, the localized heating process helps to modify the material properties of the individual devices for a short period of time, while the global heating process could be time-consuming and detrimental to the device's performance or yield. In early studies, this annealing process helped to realize the phase transition of materials from amorphous to microcrystalline^4,5^. This helps to alleviate the thermal budget for forming crystalline materials. To date, pulsed laser annealing is the next promising candidate to further relieve the thermal budget of the laser annealing process, especially for the process at the back-end-of-line (BEOL)^2,6^. Previous studies showed the possibility of improving crystalline structures of aluminum nitride (AlN)^7,8^ or zinc oxide (ZnO) nanofilms^9^ when the structural voids can be minimized with better surface morphology^10^. Meanwhile, the laser annealing technique has also been applied to dopant activation of Be-implanted gallium nitride (GaN)^11^ or silicon/germanium^12^. In addition to the applications in semiconductors, laser annealing also helps to modify the optical properties of the integrated optics devices. For instance, by pulsed and continuous laser irradiation, optical waveguides exhibit lower scattering loss by introducing more crystalline structures with refractive index change^13^ or by improving the interface quality^14^. It has also been used for realizing etch-free waveguides^15,16^ of photonic networks. Although this process is promising for integrated waveguides, the discussion in photonic devices is still limited. A few works address the potential to adjust the resonant frequencies of waveguide resonators^17,18^. However, no improvement in the quality (Q) factor is identified.

In this work, we harness femtosecond laser annealing to enhance the surface roughness and Q factor of silicon nitride (Si_3_N_4_) waveguide resonators. Compared to the previous conventional furnace annealing, our work results in several new achievements. Firstly, the surface roughness of Si_3_N_4_ waveguide can be potentially refined, validated using atomic force microscopy (AFM). Secondly, with localized thermal treatment by laser annealing, the waveguide material can be individually treated without the global heating of the device. This avoids the risk of wafer bending and stress-induced defects. Last, we successfully realize better intrinsic quality (Q) factors of the waveguide resonators using the femtosecond laser annealing technique. The intrinsic Q factor can be boosted by around 1.3 times, showing the capability to yield low-loss integrated waveguides. This also shows a strong correlation with the identified improvements in surface roughness. Based on the above demonstrations, the modification of Si_3_N_4_ waveguides shows the potential to yield high-quality integrated photonics with an efficient, low-cost, and localized annealing process for advanced fabrications.

## Waveguide resonator fabrication and optical measurement

To realize the improvement of device quality by the femtosecond laser annealing, we fabricate photonic devices with Si_3_N_4_ films and evaluate the device performance. Within the past decade, silicon photonics has gained significant attention and extensive research, owing to its remarkable versatility across diverse fields, such as optical communication, nonlinear photonics, quantum photonics, and artificial intelligence (AI)^19^. Among all on-chip photonics, integrated waveguide resonators play an important role in realizing optical functionalities, such as filters, modulators, sensors, and microwave components^20,21^. Here, we demonstrate waveguide resonators based on low-pressure chemical vapor deposition (LPCVD) Si_3_N_4_ films as the core layer. The laser annealing effect on the quality (Q) factor of waveguide resonators will be explored.

### Device fabrication

To fabricate the Si_3_N_4_ waveguide resonator, first, we deposited 500-nm LPCVD Si_3_N_4_ thin films on wafers with a 4-μm thick silicon oxide (SiO_2_) layer, which was thermally grown in a diffusion furnace. The recipe is based on the gas of dichlorosilane (DCS, SiH_2_Cl_2_) and ammonia (NH_3_) for a low-stress Si_3_N_4_ film. A Si_3_N_4_ film has been used as a hard mask for traditional CMOS fabrication processes due to the available etching selectivity both to silicon and silicon oxide^22^ or as a core layer for photonic devices due to the low optical absorption^23^. Traditionally, LPCVD-grown Si_3_N_4_ offers better surface uniformity than that of plasma-enhanced chemical vapor deposition (PECVD), and the material absorption at the telecommunication band is low due to the absence of N–H bonds at high temperatures^24^.

**Figure 1:**
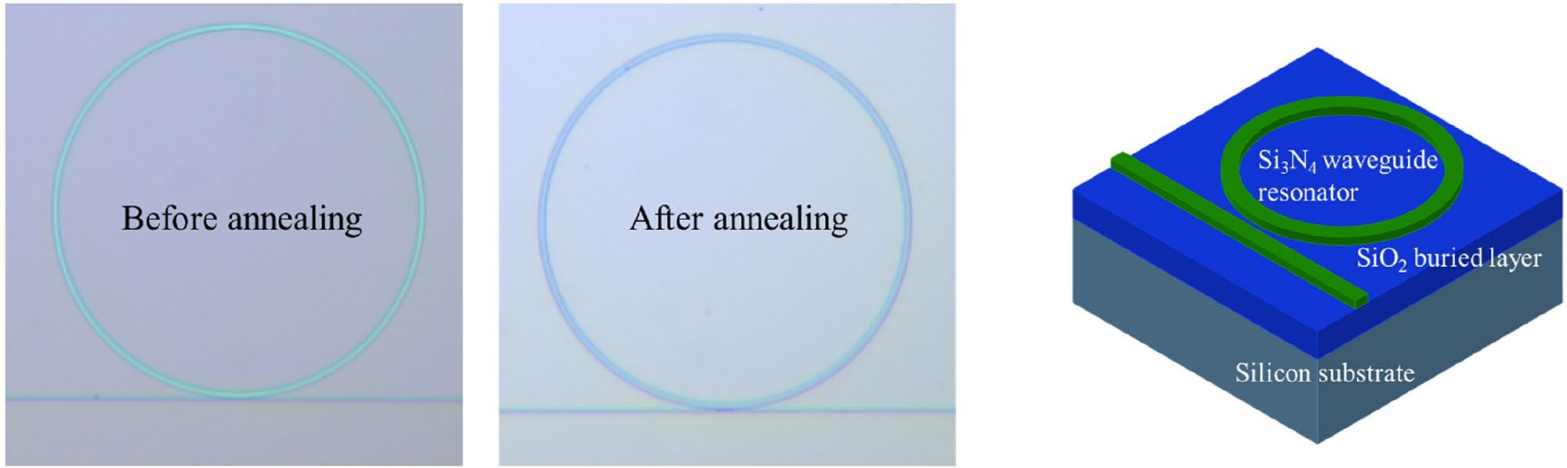
OM images and the schematic of the fabricated Si_3_N_4_ waveguides resonator with a cross-section 500 nm × 3 μm and 200-μm resonator diameter.

Then, the waveguide resonators are patterned by I-line (365 nm) stepper lithography and dry-etched in a high-density plasma etching tool. The detailed processes are described in Ref.^25^. Figure 1 shows the microscope (OM) images and the schematic of the Si_3_N_4_ waveguide resonators. The waveguide resonators are designed with cross-section 500 nm × 3 μm with a 0.4-μm gap and 200-μm diameter. To perform the laser annealing on the Si_3_N_4_ core layer, the fabricated device is air-cladded without any cladding layer^26^.

### Setup of femtosecond laser annealing

In this study, we utilized femtosecond (fs) laser pulses to refine silicon nitride (Si_3_N_4_) surfaces, a method chosen for its precise energy control and reduced thermal impact, suitable even for extensive planar areas. This technique minimizes the heat-affected zone, crucial for preserving the Si/SiO_2_ substrate beneath. Despite Si_3_N_4_'s low linear absorption at 1035 nm^27^, the femtosecond laser's intense peak power triggers efficient nonlinear absorption processes^28–30^, such as multiphoton absorption, enabling targeted surface enhancements. This approach adeptly balances surface roughness refinement with the preservation of the substrate's structural integrity.

The femtosecond laser, mRadian Kasmoro-1030, operates at a center wavelength of 1035 nm, a repetition rate of 100 kHz, and a pulse duration of 287 fs. The emitted beam is directed onto the sample surface using a 5X Mitutoyo objective, resulting in a focal spot size of 28 µm. By employing a defocusing technique, the laser beam spot size on the sample was expanded to match the 200 µm diameter of the waveguide resonators. Experiments involving laser annealing were carried out at different optical power settings. The samples were placed on a Newport ONE-XY200 motorized 2-D linear stage, facilitating movement to various positions for experimental convenience.

### Optical characterization

**Figure 2:**
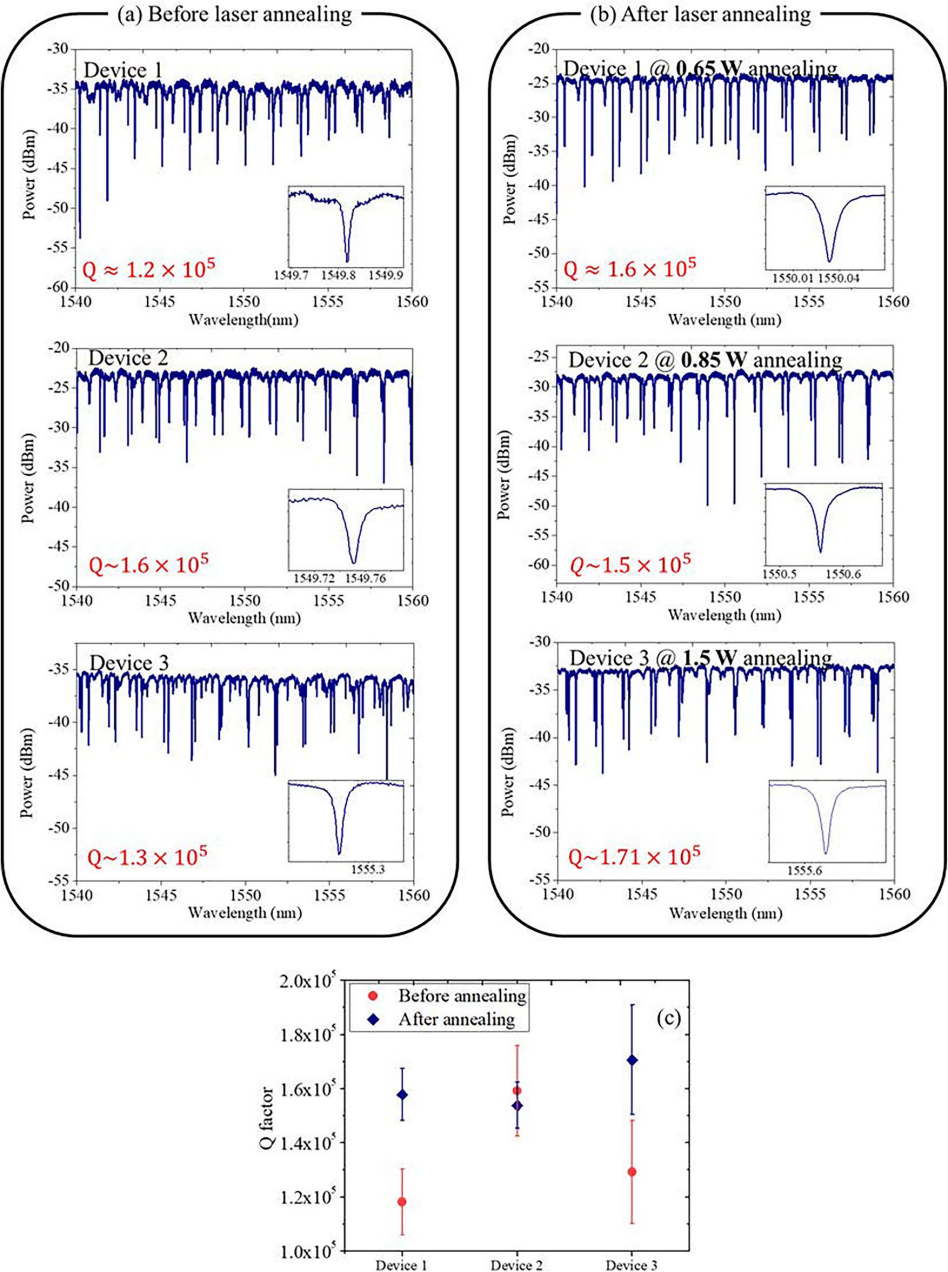
Transmission spectra of the waveguide resonators (**a**) before and (**b**) after femtosecond laser annealing with 0.65 W, 0.85 W, and 1.5 W. (**c**) Average and standard deviation of Q factors from different devices.

To assess the impact of annealing, three resonators with identical layouts on the same chip are fabricated and the corresponding intrinsic Q factors are measured before and after annealing. Optical characterization of the waveguide resonators was conducted using a tunable laser centered around a wavelength of 1550 nm for transmission assessments. This laser was coupled into the Si_3_N_4_ waveguide using a pair of lensed fibers, while the polarization was adjusted to a quasi-TE mode. Figure 2a depicts the transmission spectra of these waveguide resonators prior to femtosecond laser annealing. By fitting the cavity resonance^25^ of the observed spectra, the intrinsic Q factors can be then determined. In our pursuit of a high-Q device, a waveguide width of 3 μm is adapted to minimize the effect on sidewall roughness but it also allows the oscillation of higher-order modes. To avoid the impact of avoid-mode-crossing (AMX) between different mode families and the abrupt change of Q factors^26,31^, the evaluation of the intrinsic Q is selected out of the mode-crossing region. The demonstrated air-cladded waveguide resonators by the I-line stepper exhibit average Q factors around 1.3 × 10^5^, similar to that in Ref.^26^.

Subsequently, we applied the femtosecond laser annealing process to the three resonators with 2000 pulses at average power values of 0.65 W, 0.85 W, and 1.5 W and carried out characterization to assess its impact on the Q factors after annealing. The decision for maintaining a constant number of pulses (2000) and a fixed repetition rate (100 kHz) was strategically made to concentrate on demonstrating the impact of femtosecond pulse irradiation on Si_3_N_4_ surfaces, especially given the novelty of this application in the context of waveguide resonator enhancement. The choice to hold the number of pulses and repetition rate steady was driven by practical constraints, including the limited availability of waveguide resonators for our experiments and the intent to keep the study within a manageable scope. These parameters were selected based on preliminary tests that indicated a balance between observable annealing effects and the practicality of experimental execution.

The transmission spectra of the waveguide resonators post-annealing are depicted in Fig. 2b. Following laser annealing at 0.65 W and 1.5 W, there is an increase in the Q factor to 1.6 × 10^5^ and 1.7 × 10^5^, respectively, indicating an enhancement by approximately 1.3 times. However, for annealing at 0.85 W, where the initial Q was 1.6 × 10^5^ (higher than the others), no marked improvement was observed. In fact, the observed Q after annealing was even slightly lower. This suggests that the annealing process is particularly beneficial for resonators with initially lower Q factors, and the relationship between annealing power and Q factor improvement is not pronounced. Figure 2c further displays the average and standard deviation of Q factors for each device, revealing a slight reduction in Q factor variability. The error bars are statistically evaluated from the measured Q factors of the transmission spectra in Fig. 2a,b within the wavelength range of 1540–1560 nm, while multiple resonances are selected from the fundamental mode with a higher Q of different mode families.

**Figure 3:**
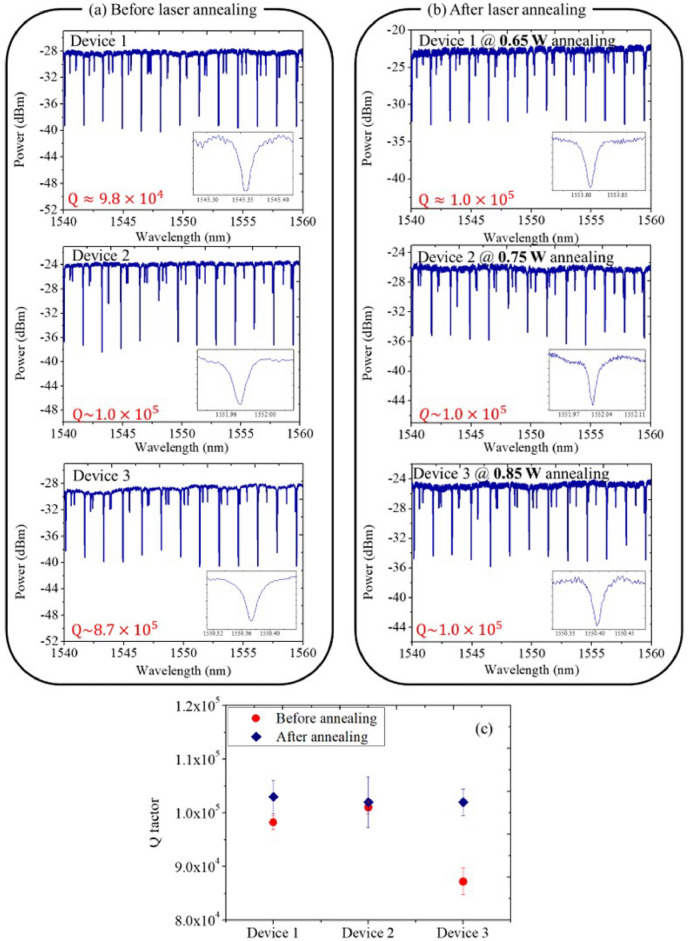
Transmission spectra of the waveguide resonators (**a**) before and (**b**) after femtosecond laser annealing with 0.65 W, 0.75 W, and 0.85 W. (**c**) Average and standard deviation of Q factors from different devices.

To further validate the effect on the Q factor, we employ another chip again with three identical resonators. However, this time, rather than being fabricated based on a spliced device, the chip was manufactured using 6-inch full-wafer processes.^32^. Figure 3a,b show the transmission spectra of the waveguide resonators before and after femtosecond laser annealing while the annealing power is set at 0.65, 0.75, and 0.85 W, respectively. Figure 3c shows the corresponding average and standard deviation of Q factors. Clearly, we can see that, for resonators that started off with relatively low Q factors, there's a notable improvement (approximately 1.3 times) in Q factors, similar to that observed in Fig. 2. Meanwhile, we observe that the power dependency on the annealing process is not critical. The slightly lower Q factors observed in comparison to those in Fig. 2 can be attributed to the utilization of a thinner buried SiO_2_ layer (2 µm) in the 6-in. full-wafer processes.

**Figure 4:**
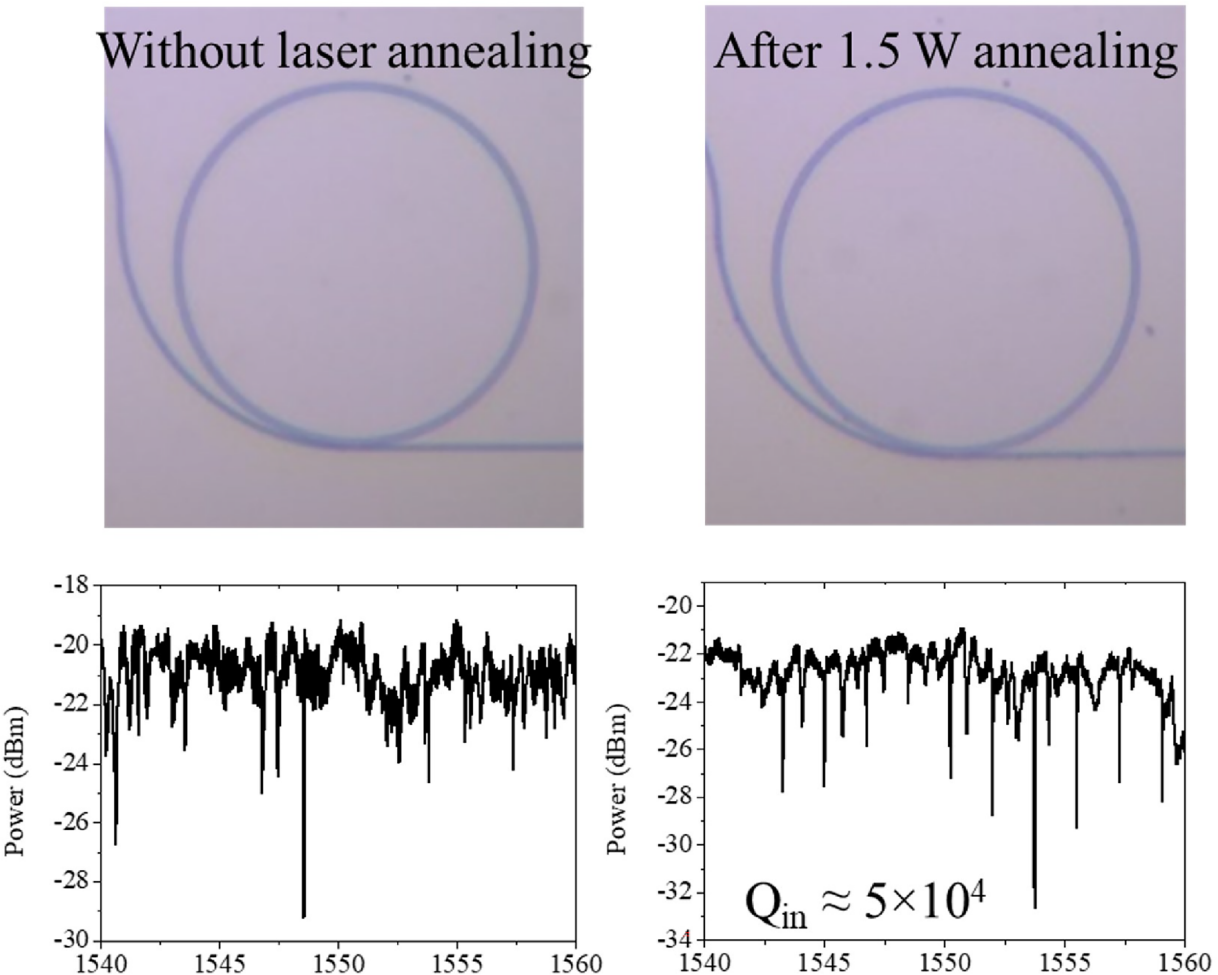
OM images and the corresponding transmission spectra before and after femtosecond laser annealing.

Additionally, we present an interesting extreme case in which no clear resonance was initially observed as shown in Fig. 4. After applying femtosecond laser annealing at 1.5 W, the treated waveguide achieved resonance conditions with a Q factor greater than 10^4^. This demonstrates that the laser annealing process could play a crucial role in fabricating functional photonics.

These outcomes align closely with the data obtained from the roughness of the annealed Si_3_N_4_ film via atomic force microscopy (AFM), discussed in subsequent sections. In essence, femtosecond laser annealing holds promise in rectifying subpar surfaces, thereby enhancing the efficacy of Si_3_N_4_-based photonics.

## Surface roughness characterization of Si_3_N_4_ film

We hypothesize that the enhancement in the Q factor is primarily attributed to the improvement in surface roughness. Our AFM measurements suggest the roughness of the Si_3_N_4_ films over a 5 µm × 5 µm area. Nonetheless, the intricate dimensions and ring-shaped design of our resonators limit evaluations to a mere 3 µm width, which presents substantial challenges for accurate roughness assessment. Additionally, the resonators' vertical waveguide sidewalls hinder the use of traditional tip-based surface profilers that are susceptible to vibration noise and tip damage when encountering sharp edges. To substantiate the effects of surface refinement, we executed femtosecond laser annealing experiments on Si_3_N_4_ films, assessing the surface roughness pre- and post-annealing. In preparation for these experiments, a 500-nm thick Si_3_N_4_ film was deposited by LPCVD at 800 °C onto a silicon substrate. This approach not only verifies the impact of surface smoothness on the Q factor but also addresses the measurement constraints posed by the specialized geometry of our resonators.

**Figure 5:**
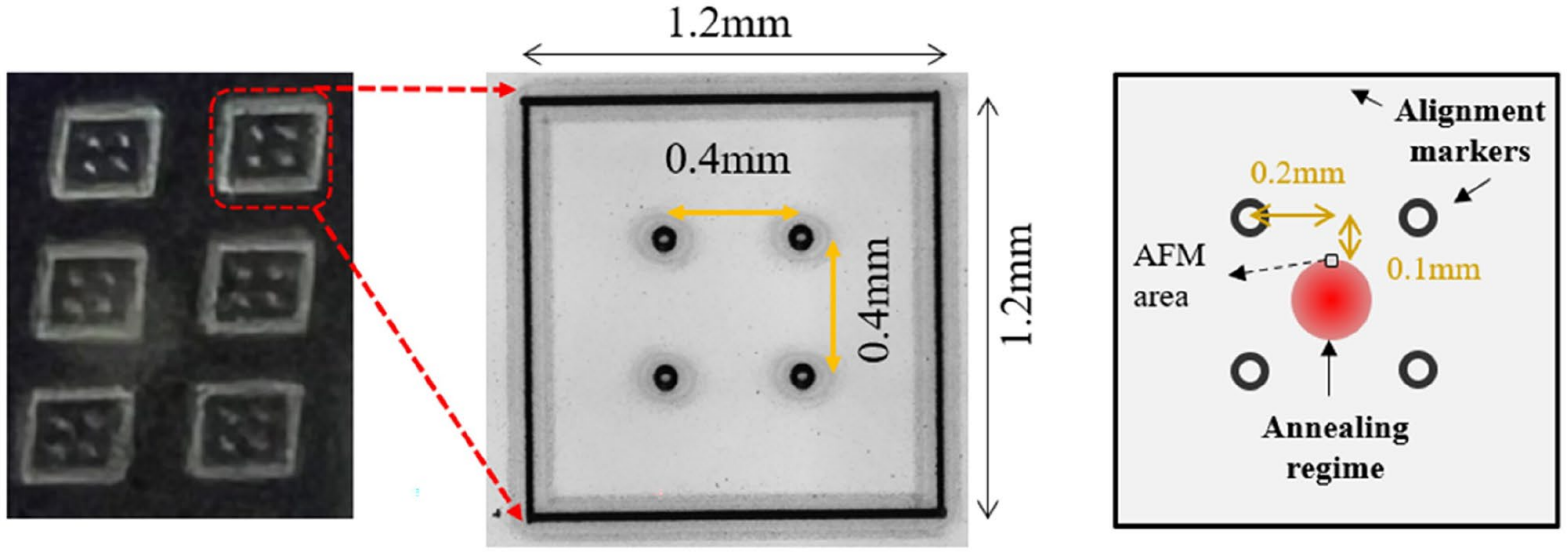
Blanket Si_3_N_4_ film with laser-patterned markers.

For precise identification of each annealing region for ensuing AFM analysis, we marked each alignment using a 0.4-W average power and a 28-µm focal spot size. Each region encompasses a 1.2 mm × 1.2 mm square, containing four internal marking spots, as depicted in Fig. 5. These markers help define the specific annealing zones. Different optical powers were then applied to anneal each region, centering a 200-µm spot size on the specified area. To elucidate the impact of femtosecond laser annealing on the surface texture of the films, we employed AFM to assess the surface profiles before and after annealing within a 5 µm × 5 µm area. It is important to note that during the annealing of the waveguide, the laser spot is positioned at the resonator's center. As a result, the area of the film examined by AFM is not centered and is depicted in Fig. 5.

### Surface morphology of Si_3_N_4_ films by atomic force microscopy (AFM)

**Figure 6:**
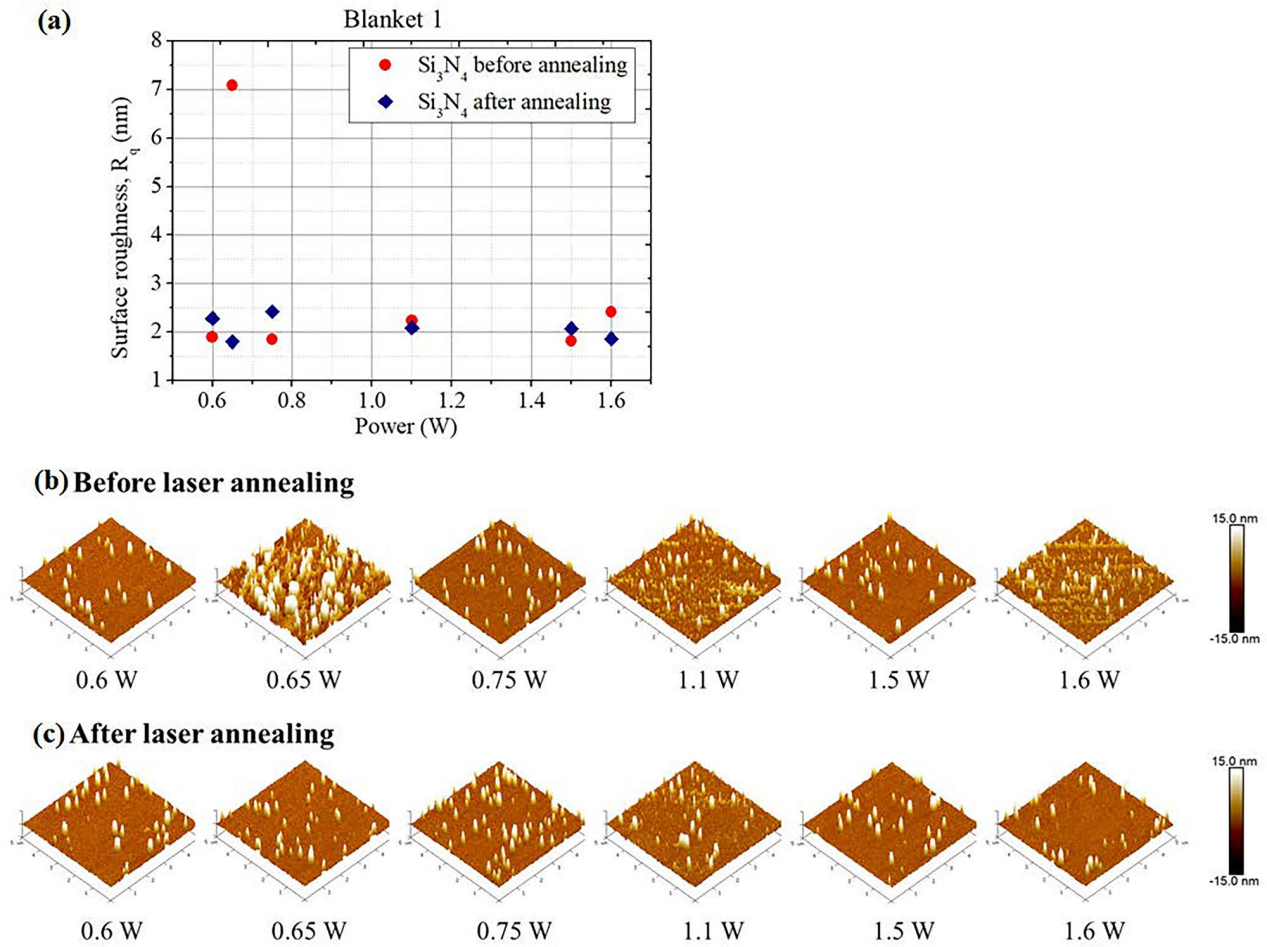
Surface roughness characterization of (**a**) root-mean-square surface roughness (R_q_) by AFM before and after the laser annealing process. The AFM images are shown (**b**) before and (**c**) after the laser annealing.

Figure 6 shows the measured root-mean-square surface roughness (R_q_) before and after the femtosecond laser annealing process. *R*_*q*_ is calculated as ${R}_{q}=\sqrt{\frac{1}{L}{\int }_{0}^{L}{Z}^{2}(x)dx}$, where *L* is the evaluation length over which the roughness is measured and Z(x) represents the surface height at each point along the length *L*, measured from the mean line. ${R}_{q}$ provides insight into the standard deviation of surface heights and capturing variations more effectively. We anneal each designated region with 2000 pulses with average optical power ranging from 0.6 to 1.6 W. First, we can observe that the average R_q_ is 2.9 nm with a standard deviation of 2.1 nm before the annealing process, showing the good quality of a LPCVD Si_3_N_4_ film. This identification agrees with the previous findings of a LPCVD Si_3_N_4_ film^33^. The corresponding AFM images of a 5 µm × 5 µm surface area before and after the laser annealing are shown in Fig. 6b,c, respectively. It is evident that the unprocessed Si_3_N_4_ film exhibits sporadic roughness, with certain regions, especially those later exposed to 0.6-W and 1.6-W laser powers, showcasing pronounced initial roughness levels of R_q_ around 7.1 nm and 2.4 nm respectively. Importantly, we can see that femtosecond laser annealing, within the specified power range, does not markedly alter the surface roughness of films that were initially of high quality. However, for films that started off with subpar surfaces, there's a notable improvement in roughness, with R_q_ values dropping from 7.1 to 1.8 nm, aligning closely with the average R_q_ of 2.9 nm. It suggests the potential to improve the poor Si_3_N_4_ surface by the femtosecond annealing. Furthermore, surfaces that were initially of high quality remained largely unaffected across different annealing powers. These observations align with the noted changes in the Q factors of the annealed waveguides, suggesting that the primary driver for the enhancement in Q-factor is likely the refinement in surface roughness.

**Figure 7:**
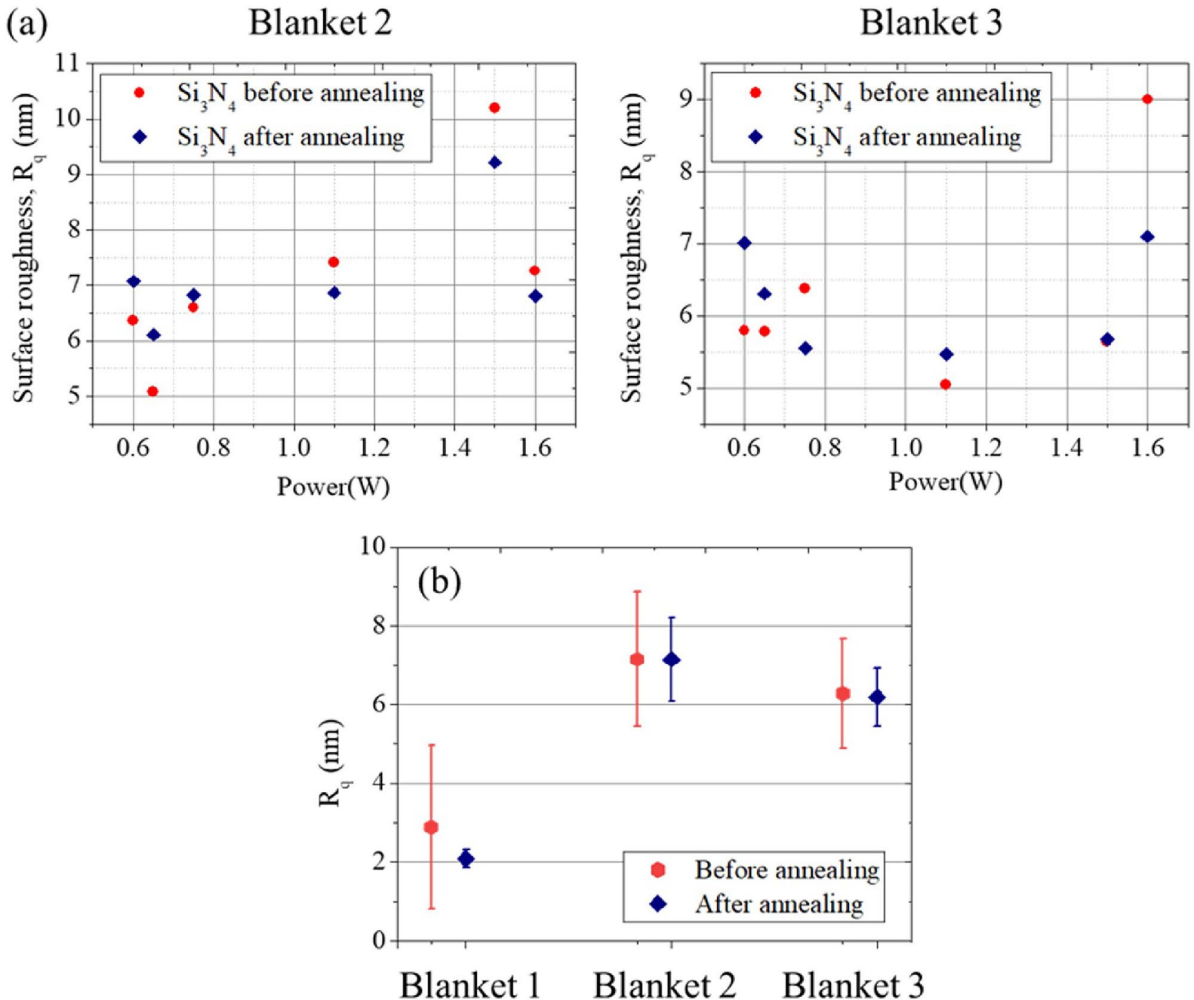
(**a**) Surface roughness (R_q_) characterization by AFM before and after the laser annealing process. (**b**) The statistics of R_q_ for three blanket Si_3_N_4_ films.

To further illustrate this idea, we measured two additional Si_3_N_4_ films and evaluated the standard deviation of the root-mean-square roughness, *R*_*q*_, with varied powers in Fig. 7. From the measurement data, the mean values of R_q_ again do not change significantly, but the standard deviation shows notable reduction. The variation of surface roughness may be resulted from the intrinsically high film stress, especially for a film thickness > 400 nm^34^. When a thick Si_3_N_4_ film is deposited, catastrophic cracking occurs, limiting the film quality. This demonstration suggests that the femtosecond laser annealing process helps to alleviate random surface roughness and provides better surface uniformity of the deposited thick Si_3_N_4_ films.

## Discussion

**Figure 8:**
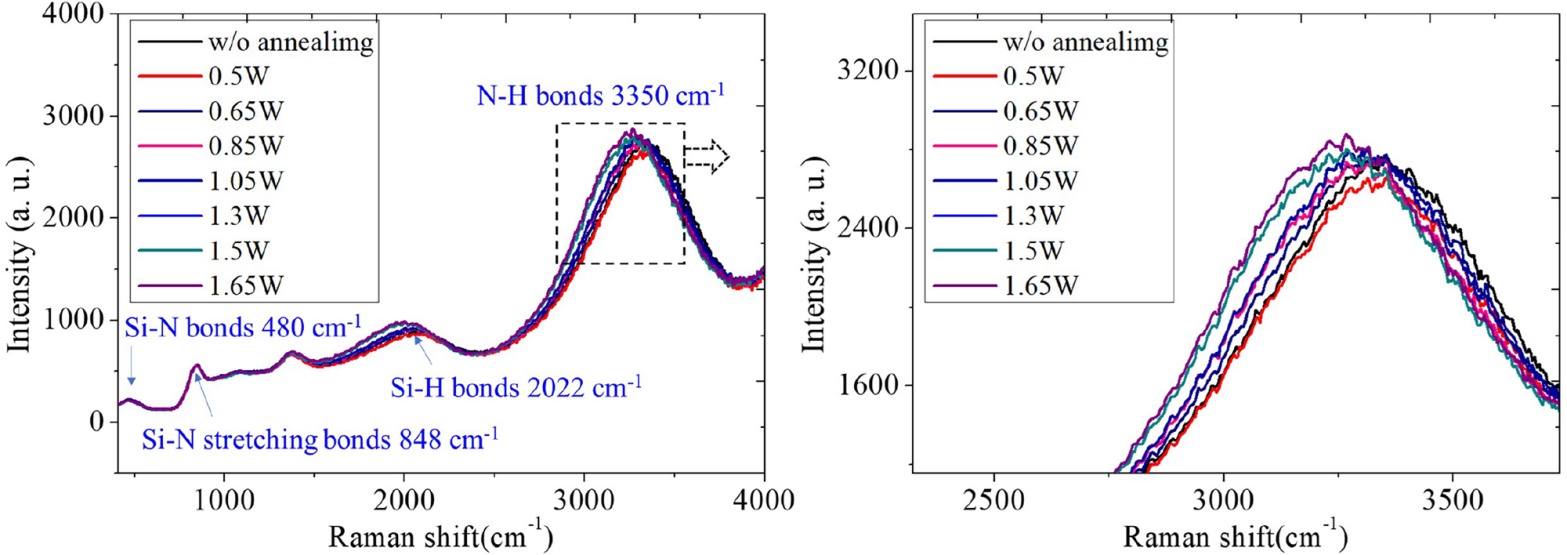
Raman spectra of the Si_3_N_4_ films with varied optical annealing powers.

In this section, we investigate the impact of femtosecond laser annealing on the composition of the LPCVD Si_3_N_4_ film. Generally, as mentioned earlier, furnace annealing is typically used to alter the material properties and improve the deposited film quality. For a Si_3_N_4_-based system, high temperatures of up to 1100–1200 °C are typically adapted to remove the residue N–H bond in Si_3_N_4_ films^34,35^. This process helps to minimize the material loss and improve the Q factors of a Si_3_N_4_ waveguide. To verify that the improvement of quality factors in our work contributes to the reduction of surface roughness instead of the composition change, we investigate the composition of Si_3_N_4_ films by Raman spectroscopy and study the spectral peaks with varied powers of femtosecond annealing. Figure 8 shows the Raman spectra of the Si_3_N_4_ films. For the data of varied annealing powers, the intensity is normalized by the Si–N stretching bond at the peak ≈ 848 cm^−1^ from the Si_3_N_4_ layer^36^. Two obvious peaks can be identified at around 2022 cm^−1^ and 3350 cm^−1^, corresponding to the Si–H bond and N–H bond, respectively^36,37^. Notably, neither the Si–H nor the N–H peaks exhibit substantial shifts across the range of annealing powers, implying that the film composition remains largely consistent post-laser annealing. Additionally, the slight red shift of both peaks can be identified by gradually increasing the annealing power, which is highly power-dependent. This will not contribute to the power-insensitive Q improvement. Finally, the absorption of Si_3_N_4_ films is typically associated with the prominent peak of the N–H bond^33,35^; from the measured data, the difference in Raman intensity between without and with the highest annealing power (1.65 W) is less than 7%. This suggests that, within this annealing power region, the composition of Si_3_N_4_ films remains almost the same as the as-deposited film.

It's important to clarify that while we used blanket films to infer the results of annealing on the resonators, this approach does not imply that changes in Q factor are primarily due to roughness alterations. The Q factor improvement is likely attributed to several other factors. The femtosecond laser annealing might enhance the crystallinity of the resonator material or reduce the presence of defects such as dislocations or internal stresses^38–40^, which typically contribute to optical scattering and energy loss. Additionally, the laser treatment may have altered the refractive index distribution within the waveguide^41^, improved interface conditions between the waveguide and its surroundings, and removed or reorganized surface or internal contaminants. These modifications could enhance optical confinement and reduce scattering losses, thereby increasing the Q factor. Moreover, the reduction in variability of surface roughness on Si_3_N_4_ films, i.e., the standard deviation of R_q_, suggests fewer peaks and valleys on the surface, which could reduce mode conversion and further contribute to improved optical quality in practical applications.

Also, the absence of significant changes in the Raman spectrum suggests that the structural and chemical compositions of the material remain unchanged. This observation underscores that while femtosecond laser annealing can enhance the optical properties by modifying physical attributes and conditions, it does not alter the fundamental molecular structure or chemical composition of the waveguide materials.

In this study, unlike previous annealing approaches using either laser or furnace methods that altered the Si_3_N_4_ composition, we achieved Q-factor enhancement by refining the surface roughness. Similar to the realization of ultra-high-Q waveguide resonators^23^, the planarization of Si_3_N_4_ films with chemical–mechanical polishing (CMP) also provides better surface roughness^42^. However, femtosecond laser annealing avoids the requirements of the time-consuming, full-wafer CMP process, and the treatment can be utilized locally. We believe the refinement effect with laser annealing can be further enhanced by properly designing the layout of waveguide resonators. For instance, using spiral structures or a wide, low-confined waveguide^43^ can effectively adjust the exposure area and power of the laser annealing to achieve the desired refinement regime.

## Summary

This study demonstrates that femtosecond laser annealing can be used to boost the Q factor in Si_3_N_4_ waveguide resonators, particularly benefiting those with initially low Q values. The enhancements may be attributed not only to the refined surface but also to reduced defects or redistribution of refractive index. Raman spectroscopy shows the stability of the material's composition after annealing, highlighting that the improvements result from physical modifications. This method offers a localized approach to enhancing photonic device performance, providing the potential for flexible photonic manufacturing. Moving forward, we plan to advance our research by developing an in-situ system for pulse-by-pulse femtosecond laser annealing. This advanced system will incorporate real-time Q factor monitoring to allow for precise, dynamic adjustments during the annealing process, based on immediate feedback from the number of laser pulses and the observed changes in Q factor. We anticipate that this approach will not only solidify the gains observed in our current studies but also enhance the reproducibility and consistency of the results across all tested devices. This strategic improvement aims to optimize the Q values uniformly, thereby elevating the performance and reliability of photonic devices fabricated using this technique.

## Data Availability

The data used in this study are available from the corresponding author upon reasonable request.

## References

[1] Bell AE (1979). Review and analysis of laser annealing. RCA Rev..

[2] Van Vechten JA, Tsu R, Saris FW (1979). Nonthermal pulsed laser annealing of Si; plasma annealing. Phys. Lett. A.

[3] Poate, J.M. & Mayer, J.W. Laser Annealing of Semiconductors; Academic Press: New York, NY, USA (1982).

[4] Miyasaka M, Stoemenos J (1999). Excimer laser annealing of amorphous and solid-phase-crystallized silicon films. J. Appl. Phys..

[5] Sakaike K, Higashi S, Murakami H, Miyazaki S (2008). Crystallization of amorphous Ge films induced by semiconductor diode laser annealing. Thin Solid Film..

[6] Huet K (2020). Pulsed laser annealing for advanced technology nodes: Modeling and calibration. Appl. Surf. Sci..

[7] Lin HK, Huang YJ (2019). Crystalline characteristics of annealed AlN films by pulsed laser treatment for solidly mounted resonator applications. BMC Chem..

[8] Szekeres A (2012). Laser technology for synthesis of AlN films: Influence of the incident laser fluence on the films microstructure. J. Phys. Conf. Ser..

[9] Kim K, Kim S, Lee SY (2012). Effect of excimer laser annealing on the properties of ZnO thin film prepared by sol-gel method. Curr. Appl. Phys..

[10] Zhou Y (2022). Improving the crystal quality of AlN films by nanosecond laser annealing. J. Manuf. Process..

[11] Wang HT, Tan LS, Chor EF (2005). Pulsed laser annealing of Be-implanted GaN. J. Appl. Phys..

[12] Cristiano F (2016). Defect evolution and dopant activation in laser annealed Si and Ge. Mater. Sci. Semicond. Process..

[13] Dutta S, Jackson HE, Boyd JT (1980). Reduction of scattering from a glass thin-film optical waveguide by CO_2_ laser annealing. Appl. Phys. Lett..

[14] Dutta S, Jackson HE, Boyd JT (1981). Scattering loss reduction in ZnO optical waveguides by laser annealing. Appl. Phys. Lett..

[15] Meany T (2015). Laser written circuits for quantum photonics. Laser Photon. Rev..

[16] Tan D, Sun X, Qiu J (2021). Femtosecond laser writing low-loss waveguides in silica glass: Highly symmetrical mode field and mechanism of refractive index change. Opt. Mater. Express.

[17] Milosevic MM (2018). Ion implantation in silicon for trimming the operating wavelength of ring resonators. IEEE J. Sel. Top. Quantum Electron..

[18] Biryukova V, Sharp GJ, Klitis C, Sorel M (2020). Trimming of silicon-on-insulator ring-resonators via localized laser annealing. Opt. Express.

[19] Thomson D (2016). Roadmap on silicon photonics. J. Opt..

[20] Rabus, D. G. Integrated Ring Resonators Springer (2007).

[21] Plourde JK, Ren CL (1981). Application of dielectric resonators in microwave components. IEEE Trans. Microw. Theory Tech..

[22] Choi, Y.-K. et al. Sub-20 nm CMOS FinFET technologies. in *Proc. International Electron Devices Meeting*. Technical Digest (Cat. No.01CH37224), 19.1.1–19.1.4 (2001).

[23] Pfeiffer MHP (2018). Ultra-smooth silicon nitride waveguides based on the Damascene reflow process: Fabrication and loss origins. Optica.

[24] Yang C, Pham J (2018). Characteristic study of silicon nitride films deposited by LPCVD and PECVD. Silicon.

[25] Wang PH, Lee TH, Huang WH (2023). Fabrication of tapered waveguides by i-line UV lithography for flexible coupling control. Opt. Express.

[26] Wang PH, Wang SP, Hou NL (2023). Flexible dispersion engineering using polymer patterning in nanophotonic waveguides. Sci. Rep..

[27] E. D. Palik, Handbook of Optical Constants of Solids, Volume** 1**, Academic Press Hand (1998).

[28] Keller WJ, Shen N, Rubenchik AM (2019). Physics of picosecond pulse laser ablation. J. Appl. Phys..

[29] Rethfeld B, Ivanov DS, Garcia ME, Anisimov SI (2017). Modelling ultrafast laser ablation. J. Phys. D: Appl. Phys..

[30] Jiang L, Wang A-D (2018). Electrons dynamics control by shaping femtosecond laser pulses in micro/nanofabrication: Modeling, method, measurement and application. Light Sci. Appl..

[31] Liu Y (2014). Investigation of mode coupling in normal-dispersion silicon nitride microresonators for Kerr frequency comb generation. Optica.

[32] Huang YK, Wang PH (2024). CMOS-compatible 6-inch wafer integration of photonic waveguides and uniformity analysis. Opt. Express.

[33] Reck K, Stergaard C, Thomsen EV, Hansen O (2011). Fusion bonding of silicon nitride surfaces. J. Micromech. Microeng..

[34] Luke K, Dutt A, Poitras CB, Lipson M (2013). Overcoming Si3N4 film stress limitations for high quality factor ring resonators. Opt. Express.

[35] Xuan Y (2016). High-Q silicon nitride microresonators exhibiting low-power frequency comb initiation. Optica.

[36] Bandet J, Despax B, Caumont M (1999). Nitrogen bonding environments and local order in hydrogenated amorphous silicon nitride films studied by Raman spectroscopy. J. Appl. Phys..

[37] Scardera G, Puzzer T, Perez-Wurfl I, Conibeer G (2008). The effects of annealing temperature on the photoluminescence from silicon nitride multilayer structures. J. Crystal Growth.

[38] Amoako G (2019). Femtosecond laser structuring of materials: A review. Appl. Phys. Res..

[39] Cao Q, Zhang J, Du J (2017). Athermal repair of nanoscale defects in optical materials using a femtosecond laser. Nanoscale..

[40] Hoppius, J.S., Bialuschewski, D. *et al*. Femtosecond laser crystallization of amorphous titanium oxide thin films. *Appl. Phys. Lett*. **113** (2018).

[41] F. Chen., J. R. de Aldana Vazques. Direct femtosecond laser writing of optical waveguides in dielectrics. in *Laser Micro-Nano-Manufacturing and 3D Microprinting*, A. Hu, ed. (2020).

[42] Yang G-R, Zhao YP (1998). XPS and AFM study of chemical mechanical polishing of silicon nitride. Thin Solid Films.

[43] Liu J (2021). High-yield, wafer-scale fabrication of ultralow-loss, dispersion-engineered silicon nitride photonic circuits. Nat. Commun..

